# Significant overlap of inflammatory and degenerative features on imaging among patients with degenerative disc disease, diffuse idiopathic skeletal hyperostosis and axial spondyloarthritis: a real-life cohort study

**DOI:** 10.1186/s13075-024-03359-w

**Published:** 2024-08-03

**Authors:** Nelly Ziade, Melanie Udod, Nikolaos Kougkas, Styliani Tsiami, Xenofon Baraliakos

**Affiliations:** 1 Hotel-Dieu De France, Saint Joseph University, Beirut, Lebanon; 2 Rheumazentrum Ruhrgebiet Herne, Ruhr-University Bochum, Claudiusstr. 45, 44649 Herne, Germany; 3 Fourth Department of Internal Medicine, Hippokration University Hospital, Aristotle University of Thessaloniki, Thessaloniki, Greece

**Keywords:** Diffuse idiopathic skeletal hyperostosis, Axial spondyloarthritis, Degenerative disc disease, Imaging, Spine, Radiography, MRI

## Abstract

**Background:**

Differentiating between degenerative disc disease (DDD), diffuse idiopathic skeletal hyperostosis (DISH), and axial spondyloarthritis (axSpA) represents a diagnostic challenge in patients with low back pain (LBP). We aimed to evaluate the distribution of inflammatory and degenerative imaging features in a real-life cohort of LBP patients referred to a tertiary university rheumatology center.

**Methods:**

In a retrospective cross-sectional analysis of patients referred for LBP, demographics, symptom information, and available imaging were collected. SpA-like changes were considered in the spine in the presence of one of the following lesions typically related to SpA: erosions, sclerosis, squaring, and syndesmophytes on conventional radiographs (CR) and bone marrow oedema (BMO), erosions, sclerosis, and fat lesions (FL) on MRI. SIJ CR were graded per New York criteria; on MRIs, SIJs were evaluated by quadrant for BMO, erosions, FL, sclerosis and ankylosis, similar to the approach used by the Berlin SIJ MRI scoring system. The final diagnosis made by the rheumatologist was the gold standard. Data were presented descriptively, by patient and by quadrant, and compared among the three diagnosis groups.

**Results:**

Among 136 referred patients, 71 had DDD, 38 DISH, and 27 axSpA; median age 62 years [IQR55-73], 63% males. On CR, SpA-like changes were significantly higher in axSpA in the lumbar (50%, vs. DDD 23%, DISH 22%), in DISH in the thoracic (28%, vs. DDD 8%, axSpA 12%), and in DDD in the cervical spine (67% vs. DISH 0%, axSpA 33%). On MRI, BMO was significantly higher in DISH in the thoracic (37%, vs. DDD 22%, axSpA 5%) and equally distributed in the lumbar spine (35-42%). FL were significantly more frequently identified in DISH and axSpA in the thoracic (56% and 52%) and DDD and axSpA in the lumbar spine (65% and 74%, respectively). Degenerative changes were frequent in the three groups. Sacroiliitis (NY criteria) was identified in 49% (axSpA 76%, DDD 48%, DISH 29%).

**Conclusion:**

A significant overlap was found among DDD, DISH, and axSpA for inflammatory and degenerative imaging features. Particularly, SpA-like spine CR features were found in one-fourth of patients with DISH, and MRI BMO was found in one-third of those patients.

**Supplementary Information:**

The online version contains supplementary material available at 10.1186/s13075-024-03359-w.

## Introduction

Low back pain (LBP) is a highly prevalent condition, affecting 619 million people in 2020, nearly 10% of the world’s population, according to the Global Burden of Disease study, with a substantial societal and economic burden worldwide [1, 2].

An appropriate distinction between LBP etiologies is essential for establishing a proper management plan for the individual patient and allocating resources efficiently for the general population. In particular, differentiating between degenerative disc disease (DDD), diffuse idiopathic hyperostosis (DISH), and axial spondyloarthritis (axSpA) may represent a significant diagnostic and therapeutic challenge in rheumatological clinical practice.

As DDD is observed in most individuals increasingly with age and acknowledged as an expected feature of ageing, DISH is considered a non-inflammatory condition that involves exuberant calcification and ossification of the spinal ligaments and entheses and was first described by Forestier and Rotes-Querol in 1950 [3], although its origin was traced back to the Royal Egyptian mummies from the 15th century BC [4]. DISH mainly, but not exclusively, affects adults older than 45 years and is associated with various metabolic disorders, such as obesity, hypertension, diabetes mellitus and metabolic syndrome in general [5, 6]. Traditionally, DISH is defined according to the Resnik radiographic criteria, which require flowing spondylophytes in four contiguous vertebrae in the thoracic spine [7], relative preservation of the intervertebral disc space, and absence of apophyseal joint ankylosis and sacroiliac joint (SIJ) erosion, sclerosis, or intraarticular osseous fusion. However, several definitions were subsequently utilized to identify earlier forms of the disease and accommodate possible SIJ lesions [8, 9].

In contrast, axSpA is a chronic inflammatory immune-mediated disease, starting in young adulthood, with inflammatory back pain as the main clinical feature [10] and an association with the Human Leucocyte Antigen B27 (HLA-B27). The earliest and most characteristic findings occur in the SIJs and include inflammatory lesions (depicted by magnetic resonance imaging (MRI)) but also chronic structural changes such as fat lesions (FL), periarticular bony erosions, sclerosis and new bone formation or ankylosis. The disease later extends ascendingly to the spine, with similar findings to the SIJ, resulting in the formation of syndesmophytes, which are vertical bony bridges joining adjacent vertebral bodies anteriorly and laterally to form a bamboo-spine [11].

Although DDD, DISH, and axSpA are all associated with spinal ossification, the underlying pathophysiology, imaging pattern, and subsequent therapeutic targets are fundamentally different [12]. [13].

The co-existence of inflammatory and degenerative changes (DC) is problematic with increasing age, making the distinction between the degenerative and inflammatory causes of pain and disability particularly challenging [14–17].

Realizing the challenge of imaging description to establish an accurate diagnosis and its subsequent impact on management, the objective of the study was to evaluate the distribution of spinal inflammatory and degenerative imaging features in a real-life cohort of patients with chronic back pain referred to a tertiary university rheumatology center and identify those associated with DDD, DISH or axSpA.

## Methods

### Study design

This was a retrospective cross-sectional analysis of patients with the diagnosis of DDD, DISH or axSpA who were referred to a tertiary specialized university rheumatology center in the period of 2014 to 2020 (Rheumazentrum Ruhrgebiet Herne, Germany). Inclusion criteria included patients with low back pain referred for clinical suspicion of axSpA or for the purpose of rejection of this diagnosis, with available spine or SIJ imaging (CR or MRI) at the time of study inclusion. Imaging of the axial skeleton during the treatment period was based on a clinical indication arising from the corresponding symptoms.

### Variables

Demographic (age, sex, comorbidities (hypertension, diabetes, body mass index (BMI), smoking (current, ever, pack-years) and disease data (pain on a numerical rating scale (NRS), Bath Ankylosing Spondylitis Disease Activity Index (BASDAI), Bath Ankylosing Spondylitis Functional Index (BASFI), association with other rheumatic diseases (rheumatoid arthritis (RA), psoriatic arthritis (PsA), psoriasis) were collected for all patients as per the center’s usual procedures. Also, laboratory data were collected (Erythrocyte Sedimentation rate (ESR), C-reactive protein (CRP), HLA-B27, uric acid, alkaline phosphatases, HbA1c). In addition, anti-rheumatic and other drugs were recorded (non-steroidal anti-inflammatory drugs (NSAIDs) prednisone, conventional synthetic disease-modifying anti-rheumatic drugs (csDMARDs), biological DMARDs (bDMARDs), aspirin, cholesterol-lowering agents, anti-hypertensive drugs, oral antidiabetics and insulin.

The final diagnosis made by the rheumatologist (DDD, DISH and axSpA) was the gold standard.

### Data measurement and bias assessment

All images were performed based on standardized procedures [18] and scored according to previously described methods [19–24]. In brief, MRIs of the spine were available in STIR, T2- and T1-weighted sequences in the lateral view. CR of the spine were also available in the lateral view. MRIs of the SIJ were available in the semi-coronal and semi-axial view, while CR of the SIJ was available in the anteroposterior view. All images were evaluated by two independent trained readers, blinded to the final diagnosis. Discrepancies were solved by consensus together with an adjudicator. For analysis, the evaluation of all lesions where agreement was found between readers (in the independent reading or after consensus) was taken into account.

In CR of the spine, the number of quadrants with erosions, sclerosis, squaring, syndesmophytes, bridging syndesmophytes (SpA-like changes), spondylophytes, and bridging spondylophytes (degenerative changes) were evaluated in all segments (cervical C2-T1, thoracic T1-L1 and lumbar spine L1-S1). A patient was considered to have SpA-like changes in the presence of one of the following lesions typically related to SpA: erosions, sclerosis, squaring, and syndesmophytes. In addition, the percentage of patients with SpA-like changes and DC was recorded by disease group. On spinal MRI, the cervical, thoracic, and lumbar spine were evaluated by quadrant and on the patient level for BMO, erosions, sclerosis, and FL. A patient was considered to have SpA-like changes in the presence of one of these four lesions typically related to SpA according the ASAS-MRI definition [24].

In addition, disc units and patients were evaluated for Modic changes, Pfirrmann changes, and disc protrusion (DP) [21, 22]. For the spine, lesions were recorded if they were located at the vertebral corners, while for the SIJ, they had to be in the middle, cartilaginous part of the joint. In case of disc degeneration or cases of doubt as to the origin of the lesion, the lesions were considered degenerative and were evaluated accordingly. Modic lesions and Pfirrmann were evaluated according to their original definitions [21, 22].

CR of the SIJs were scored from 0 (normal) to 4 (ankylosis) according to the grading used in the modified New York criteria [23]. The presence of sacroiliitis was defined by having at least a grade 2 bilaterally or grade 3 unilaterally.

On MRI, SIJs were evaluated by quadrant for BMO, erosions, FL, sclerosis and ankylosis, similar to the approach used by the Berlin SIJ MRI scoring system [25], degenerative capsular ankylosis was also recorded. As in the spine, a patient was considered to have SpA-like changes in the presence of one of these lesions typically related to SpA, according to the ASAS MRI definition [20].

### Statistical methods

Data are presented descriptively, using frequencies and percentages for categorical variables. According to the distribution of the variables, quantitative data have been expressed as mean and standard deviation (SD), or median and interquartile range (IQR).

Data were compared among the three diagnosis groups (DDD, DISH, and axSpA). The comparison of the variables among the three groups was performed using χ2, Mann–Whitney-U and Kruskal-Wallis tests as appropriate.

There was no imputation of missing data. Statistical analyses were performed using SPSS version 25.0 (IBM Corp., Armonk, NY, USA), with a significance threshold (p-value) of less than 0.05.

### Study reporting

The study reporting complied with the Strengthening the Reporting of Observational Studies in Epidemiology (STROBE) Statement [26].

## Results

Among 136 patients, 71 were diagnosed with DDD, 38 with DISH, and 27 with axSpA. Their median age was 61.5 years [IQR 55.0-72.7] (*p* = 0.067 among the three groups, older patients in DDD (64.0 years [56.0–73.0]) compared to axSpA (56.0 [51.0–64.0]), *p* = 0.020); 86 (63.2%) were males (higher proportion of males in the axSpA group (24/27 (88.9%)), compared to both DDD (38/71 (53.5%)) and DISH (24/38 (63.2%)), *p* = 0.003) (Table 1).

**Table 1:** Patient characteristics in the three spinal diagnosis groups (percentages are presented in columns, *p*-value indicates the comparison between the three groups)

	Degenerative Spine Disease	DISH	Axial Spondyloarthritis	All	*p*-value
N	71	38	27	136	
Age in years, median [IQR]	64.0 [56.0–73.0]^@3^	61.5 [54.7–72.7]	56.0 [51.0–64.0]	61.5 [55.0-72.7]	0.067
Male Gender, N (%)	38 (53.5)	24 (63.2)	*24 (88.9)* ^##^	86 (63.2)	0.003
BMI category, N (%)					
- Normal	3 (4.2)	4 (10.5)	4 (14.8)	11 (8.1)	0.439
- Overweight	21 (29.6)	8 (21.1)	7 (25.9)	36 (26.5)	
- Obese	36 (50.7)	18 (47.4)	14 (51.9)	68 (50.0)	
- Morbidly obese	11 (15.5)	8 (21.1)	2 (7.4)	21 (15.4)	
Current smoker, N (%)	10 (14.5)	7 (18.4)	*14 (53.8)* ^*##*^	31 (23.5)	< 0.001
Ever smoker, N (%)	25 (36.2)	13 (34.2)	*19 (73.1)* ^*##*^	57 (42.9)	0.002
Smoking, pack-years, mean [SD]	4.2 [11.4]	3.4 [11.6]	*15.1 [19.3]* ^*##*^	5.9 [13.6]	0.008
Hypertension, N (%)	*53 (82.8)* ^*@3*^	26 (72.2)* ^*3*^	12 (48.0)	91 (72.8)	0.004
Diabetes, N (%)	*20 (29.0)* ^*@3*^	15 (39.5) * ^*3*^	2 (7.4)	37 (27.6)	0.012
Other RMDs, N (%)					
- Rheumatoid Arthritis	15 (21.1)	7 (18.4)	0 (0)	22 (16.1)	0.061
- Psoriatic Arthritis	7 (9.4)	1 (3.7)	2 (5.3)	10 (7.3)	
- Psoriasis	3 (4.2)	0 (0)	0 (0)	3 (2.2)	
Aspirin, N (%)	28 (40.0)^@2^	8 (21.1)	6 (22.2)	42 (31.1)	0.069
Cholesterol-lowering agents, N (%)	24 (34.3)^@3^	9 (23.7)	3 (11.1)	36 (26.7)	0.059
Anti-hypertensive, N (%)	58 (82.9)^@3^	28 (73.7)	13 (48.1)	99 (73.3)	0.002
Oral antidiabetics, N (%)	*19 (27.5)*	12 (33.3)* ^*3*^	2 (7.4)	33 (25.0)	0.039
Insulin, N (%)	7 (10.0)	6 (16.2)*^3^	0 (0)	13 (9.7)	0.080
BASFI, mean [SD]	4.1 [3.8]	5.0 [3.0]	5.4 [2.8]	5.0 [3.0]	0.721
BASDAI, mean [SD]	2.1 [3.2]	4.6 [3.2]	5.3 [1.6]^#1^	4.6 [2.4]	0.060
NRS pain, mean [SD]	7.2 [1.8]	7.1 [1.9]	7.0 [2.2]	7.1 [2.0]	0.905
HLA-B27, N (%)	7/23 (30.4)	4/18 (22.0)	*14/24 (58.3)* ^*#2*^	25/65 (38.5)	0.040
ESR mm/h, mean [SD]	19.1 [16.4]^@3^	15.8 [11.9]	12.4 [11.0]	17.3 [14.6]	0.063
CRP mg/dl, mean [SD]	0.9 [1.2]	1.2 [2.9]	0.6 [0.7]	0.9 [1.8]	0.343
High CRP, N (%)	30 (43.5)	14 (36.8)	8 (29.6)	52 (38.8)	0.447
HbA1c %, mean [SD]	*6.4 [0.9]* ^*@3*^	*6.7 [1.0]* ^*@3*^	5.6 [0.8]	6.4 [1.0]	0.031
Uric Acid mg/dl, mean [SD]	6.0 [1.6]	6.0 [1.7]	6.4 [1.8]	6.1 [1.6]	0.580
Alkaline phosphatases IU/ml, mean [SD]	81.1 [22.8]	88.7 [26.0]	86.3 [24.3]	84.4 [24.1]	0.223
Treatment at discharge, N (%)					
- NSAIDs	31 (44.3)	18 (47.4)	*20 (74.1)* ^*##*^	69 (51.1)	*0.027*
- Glucocorticoids	17 (24.3)	11 (28.9)	3 (11.1)	31 (23.0)	0.222
- csDMARDs	17 (24.3)	7 (18.4)	5 (18.5)	29 (21.5)	0.756
- bDMARDs	3 (4.3)	1 (2.6)	3 (11.1)	7 (5.2)	0.340

BASDAI: Bath Ankylosing Spondylitis Disease Activity Index, BASFI: Bath Ankylosing Spondylitis Functional Index, bDMARDs: biological Disease-Modifying Anti-Rheumatic Drugs, BMI: Body Mass Index, CRP: C-reactive Protein, csDMARDs: conventional synthetic Disease-Modifying Anti-Rheumatic Drugs, DC: Degenerative Changes, DISH: Diffuse Idiopathic Skeletal Hyperostosis, ESR: Erythrocyte Sedimentation Rate, HLA-B27: Human Leucocyte Antigen B27, IQR: Inter-Quartile Range, NRS: Numerical rating Scale, NSAIDs: Non-Steroidal Anti-Inflammatory Drugs, axSpA: axial Spondyloarthritis, SD: Standard Deviation

The p-value is related to the simultaneous comparison between the three groups

A p-value < 0.05 was considered as significant

Italic numbers indicates significant two by two-by-two differences:

^##^ Significantly higher in axSpA versus DISH and Degenerative Disc Disease

^#1^ Significantly higher in axSpA versus Degenerative Disc Disease

^#2^ Significantly higher in axSpA versus DISH

*^3^ Significantly higher in DISH versus axSpA

^@2^ Significantly higher in Degenerative Disc Disease versus DISH

^@3^ Significantly higher in Degenerative Disc Disease versus axSpA

BASDAI was available for 25 patients (5 with DISH, 4 with DDD, and 16 with axSpA), BASFI for 25 patients (5 with DISH, 5 with DDD, and 15 with axSpA), HbA1c for 50 patients (16 with DISH, 26 with DDD, and 8 with axSpA), and HLA-B27 for 65 patients (18 with DISH, 23 with DDD, and 24 with axSpA)

Patients had high levels of comorbidities: 91 (72.8%) had hypertension (higher in the DDD (82.8%) and DISH (72.2%) groups compared to axSpA (48%), *p* = 0.004). Also, 37 (27.6%) had diabetes, with a higher prevalence in DISH (39.5%) and DDD (29.0%) groups, compared to axSpA 7.4% (*p* = 0.012), also reflected in higher levels of HbA1c and treatment with oral antidiabetics and insulin. In addition, 65.4% of patients were classified as obese or morbidly obese (*p* = 0.439 among the three groups). Conversely, 31 (23.5%) were current smokers, more in the axSpA group (53.8%), compared to DISH (18.4%) and DDD (14.5%), *p* < 0.001 (Table 1).

**Fig. 1:**
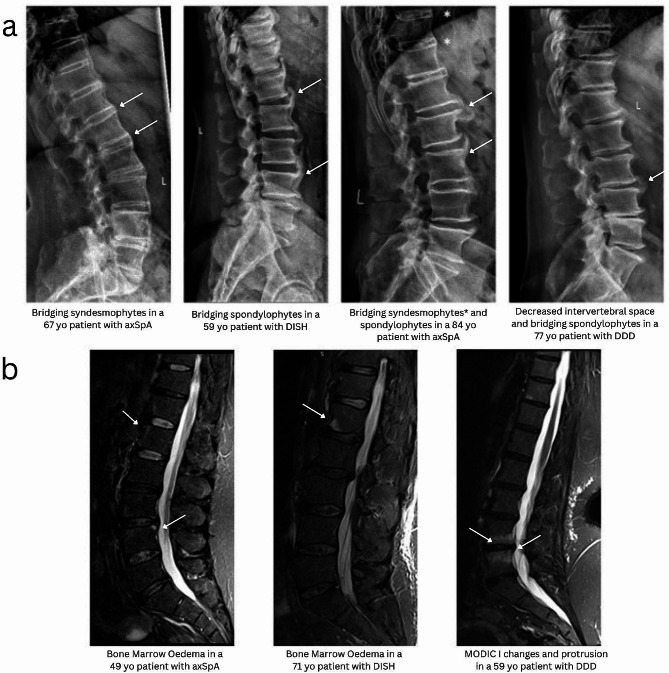
Sample imaging of spinal lesions: **a** Sample CRs of spinal lesions in patients with axSpA, DISH, and DDD. **b** Sample MRIs of spinal lesions in patients with axSpA, DISH, and DDD. axSpA: axial Spondyloarthritis, CR: Conventional Radiography, DDD: Degenerative Disc Disease, DISH: Diffuse Idiopathic Skeletal Hyperostosis, MRI: Magnetic resonance Imaging. Characteristic lesions were indicated by arrows

Typical examples of inflammatory and degenerative changes on spinal imaging (X-rays and MRIs), including overlapping features, are shown in Fig. 1.

### Availability of the imaging data

Since the imaging tests were performed according to the clinical indication, they were available for a variable proportion of patients. For the CR, cervical spine data was available for 20 patients, thoracic for spine for 128 patients, lumbar spine for 116 patients, and SIJ for 117 patients. As for the MRIs, cervical spine data was available for 27 patients, thoracic spine for 102 patients, lumbar spine for 95 patients and SIJ for 30 patients.

### Spine X-rays

**Fig. 2:**
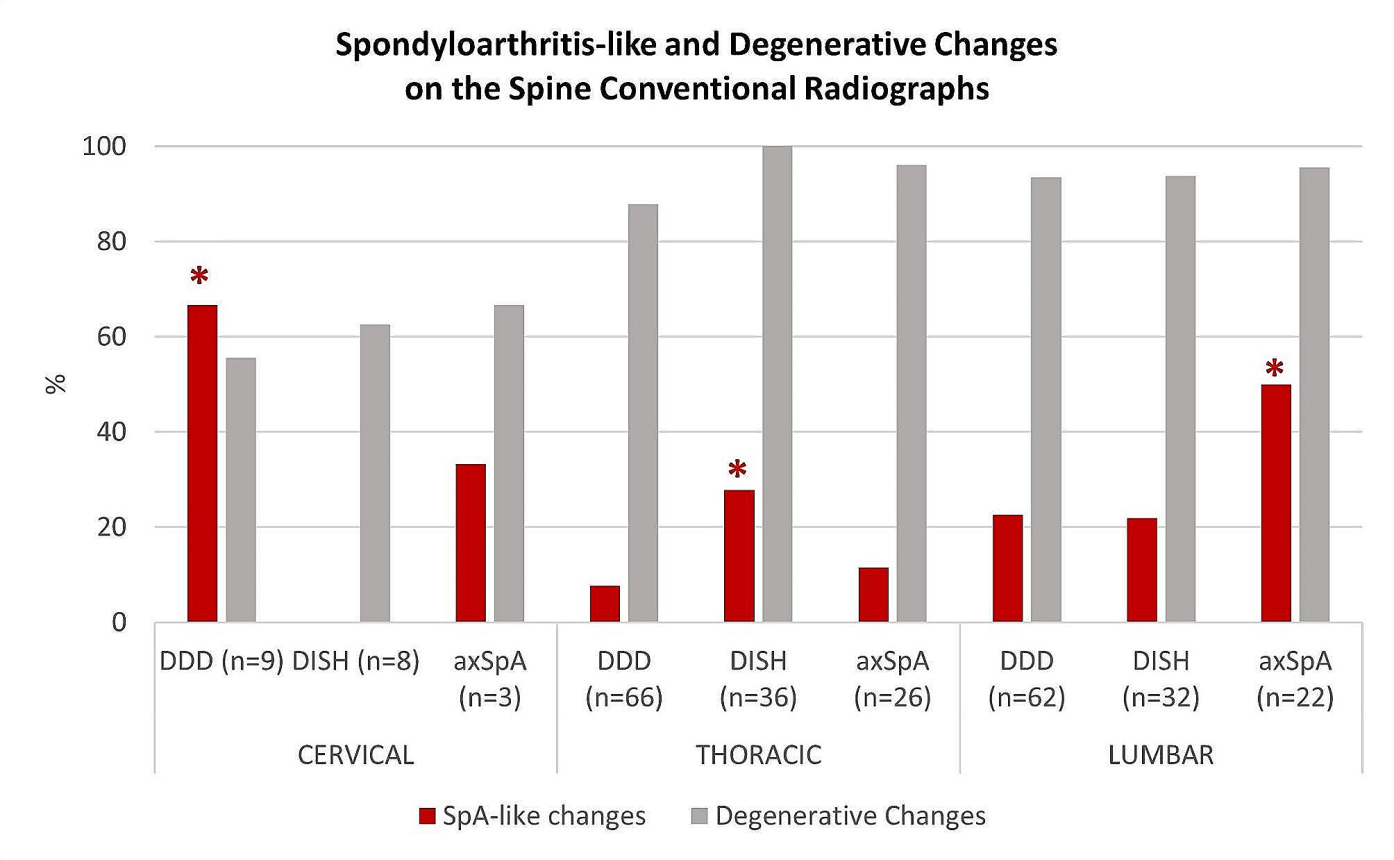
SpA-like changes in the spine CR in patients with DDD, DISH, and axSpA. The presence of SpA changes was defined by a positive score for erosions, sclerosis, squaring, syndesmophytes, or bridging syndesmophytes; the presence of degenerative changes was defined by the presence of spondylophytes or bridging spondylophytes. axSpA: axial Spondyloarthritis, CR: Conventional radiography, DDD: Degenerative Disc Disease, DISH: Diffuse Idiopathic Skeletal Hyperostosis, SpA: Spondyloarthritis. *Statistically significant difference (higher prevalence of SpA-like changes): DDD vs. DISH in cervical, DISH vs. DDD in thoracic, axSpA vs. DISH and DDD in lumbar spine

SpA-like changes were prevalent in the three groups (Table 2; Fig. 2). In the cervical spine, they were identified in 7/20 (35%) patients: significantly more in DDD 6/9 (66.7%) compared to DISH (0/8), and in 1/3 (33.3%) of axSpA (*p* = 0.013). In the thoracic spine, SpA-like changes were identified in 18/128 patients (14.1%): significantly more in DISH (10/36 (27.8%)) compared to axSpA (3/26 (11.5%)) and DDD (5/66 (7.6%)), *p* = 0.024. In the lumbar spine, they were identified in 32/116 (27.6%) patients: significantly more in axSpA (11/22 (50%)) compared to both DDD (14/62 (22.6%)) and DISH (7/32 (21.9%)), *p* = 0.033.

**Table 2:** Spine X-ray lesions (A: Cervical, B: Thoracic, C: Lumbar; the presence of SpA changes was defined by a positive score for erosions, sclerosis, squaring, syndesmophytes, or bridging syndesmophytes; the presence of degenerative changes was defined by the presence of spondylophytes or bridging spondylophytes)

	Degenerative disc disease	DISH	Axial spondyloarthritis	All	*p*-value
**A: Cervical Spine X-ray lesions**					
**Number of patients evaluated**	9	8	3	20	
**Number of disco-vertebral units evaluated**	45	27	7	79	
**Number of quadrants evaluated**	**180**	**108**	**28**	**316**	
**Erosions**					
Quadrants with SpA erosions, N (%)	11 (6.1)	0	1 (3.5)	12 (3.8%)	
Patients with SpA erosions, N (%)	5 (55.6)	0	1 (33.3)	6 (30.0)	0.277
Quadrants with erosions per patient	2.2		1	2	
**Sclerosis**					
Quadrants with SpA sclerosis, N (%)	0	0	0	0	
Patients with SpA sclerosis, N (%)	0	0	0	0	N/A
Quadrants with sclerosis per patient					
**Squaring**					
Quadrants with SpA squaring, N (%)	0	0	0	0	
Patients with SpA squaring, N (%)	0	0	0	0	N/A
Quadrants with squaring per patient					
**Syndesmophytes**					
Quadrants with SpA syndesmophytes, N (%)	5 (2,8)	0	0	5 (1,6)	
Patients with SpA syndesmophytes, N (%)	2 (2.22)	0	0	2 (10.0)	0.706
Quadrants with syndesmophytes per patient	2.5			2.5	
**Bridging syndesmophytes**					
Quadrants with SpA bridging syndesmophytes, N (%)	0	0	0	0	
Patients with SpA bridging syndesmophytes, N (%)	0	0	0	0	N/A
Quadrants with bridging syndesmophytes per patient					
Patients with SpA-like changes, N / All patients in the group (%)	*6/9 (66.7)* ^*@2*^	0/8 (0)	1/3 (33.3)	7/20 (35.0)	0.013
**Spondylophytes**					
Quadrants with spondylophytes, N (%)	39 (21.7)	10 (9.3)	9 (32.1)	58 (18.3)	
Patients with SpA spondylophytes, N (%)	5 (55.6)	4 (50.0)	2 (66.6)	11 (55.0)	0.913
Quadrants with spondylophytes per patient	7.8	2.5	4.5	5.3	
**Bridging spondylophytes**					
Quadrants with bridging spondylophytes, N (%)	10 (5.5)	24 (22.2)	4 (14.3)	38 (12.0)	
Patients with SpA bridging spondylophytes, N (%)	5 (55.6)	4 (50.0)	1 (33.3)	10 (50.0)	0.686
Quadrants with bridging spondylophytes per patient	2	6	4	3.8	
Patients with degenerative changes N / All patients in the group (%)	5/9 (55.6)	5/8 (62.5)	2/3 (66.7)	12/20 (60.0)	1.000
**B: Thoracic Spine X-ray lesions**					
**Number of patients evaluated**	**66**	**36**	**26**	**128**	
**Number of disco-vertebral units evaluated**	**224**	**172**	**93**	**490**	
**Number of quadrants evaluated**	**896**	**685**	**372**	**1953**	
**Erosions**					
Quadrants with SpA erosions, N (%)	2 (0.22)	6 (0.88)	1 (0.27)	9 (0.46)	
Patients with SpA erosions, N (%)	2 (3.0)	6 (16.7)* ^*1*^	1 (3.8)	9 (7.0)	0.038
Quadrants with erosions per patient	1	1	1	1	
**Sclerosis**					
Quadrants with SpA sclerosis, N (%)	0	0	0	0	
Patients with SpA sclerosis, N (%)	0	0	0	0	N/A
Quadrants with sclerosis per patient					
**Squaring**					
Quadrants with SpA squaring, N (%)	0	0	0	0	
Patients with SpA squaring, N (%)	0	0	0	0	N/A
Quadrants with squaring per patient					
**Syndesmophytes**					
Quadrants with SpA syndesmophytes, N (%)	0	2 (0.29)	0	2 (0.10)	
Patients with SpA syndesmophytes, N (%)	0	1 (2.8)	0	1 (0.78)	0.478
Quadrants with syndesmophytes per patient		2		2	
**Bridging syndesmophytes**					
Quadrants with SpA bridging syndesmophytes, N (%)	2 (0.22)	12 (1.8)	6 (1.61)	20 (1.02)	
Patients with SpA bridging syndesmophytes, N (%)	1 (1.5)	3 (8.3)	2 (7.7)	6 (4.7)	0.154
Quadrants with bridging syndesmophytes per patient	2	4	3	3.3	
Patients with SpA-like changes, N / All patients in the group (%)	5/66 (7.6)	10/36 (27.8)* ^*1*^	3/26 (11.5)	18/128 (14.1)	0.024
**Spondylophytes**					
Quadrants with spondylophytes, N (%)	176 (19.6)	121 (17.7)	66 (17.7)	363 (18.6)	
Patients with SpA spondylophytes, N (%)	47 (71.2)	28 (77.8)	18 (69.2)	93 (72.6)	0.709
Quadrants with spondylophytes per patient	3.7	4.3	3.7	3.9	
**Bridging spondylophytes**					
Quadrants with bridging spondylophytes, N (%)	118 (13.2)	182 (26.6)	98 (26.3)	398 (20.4)	
Patients with SpA bridging spondylophytes, N (%)	33 (50.0)	32 (88.9)* ^*1*^	*19 (73.1)* ^*#1*^	84 (65.6)	< 0.001
Quadrants with bridging spondylophytes per patient	3.6	5.7	5.2	4.7	
Patients with degenerative changes N / All patients in the group (%)	58/66 (87.9)	36/36 (100)*^1^	25/26 (96.1)	119/128 (93.0)	0.055
**C: Lumbar Spine X-ray lesions**					
**Number of patients evaluated**	**62**	**32**	**22**	**116**	
**Number of disco-vertebral units evaluated**	**305**	**141**	**127**	**573**	
**Number of quadrants evaluated**	**1218**	**563**	**505**	**2286**	
**Erosions**					
Quadrants with SpA erosions, N (%)	17 (1.4)	8 (1.4)	7 (1.4)	32 (1.4)	
Patients with SpA erosions, N (%)	12 (19.3)	6 (18.7)	5 (22.7)	23 (19.8)	0.959
Quadrants with erosions per patient	1.4	1.3	1.4	1.4	
**Sclerosis**					
Quadrants with SpA sclerosis, N (%)	1 (0.08)	1 (0.18)	2 (0.40)	4 (0.17)	
Patients with SpA sclerosis, N (%)	1 (1.6)	1 (3.1)	2 (9.1)	4 (3.4)	0.242
Quadrants with sclerosis per patient	1	1	1	1	
**Squaring**					
Quadrants with SpA squaring, N (%)	0	0	0	0	
Patients with SpA squaring, N (%)	0	0	0	0	N/A
Quadrants with squaring per patient					
**Syndesmophytes**					
Quadrants with SpA syndesmophytes, N (%)	4 (0.33)	0	1 (0.20)	5 (0.22)	
Patients with SpA syndesmophytes, N (%)	3 (4.8)	0	1 (4.5)	4 (3.4)	0.526
Quadrants with syndesmophytes per patient	1.3		1	1.2	
**Bridging syndesmophytes**					
Quadrants with SpA bridging syndesmophytes, N (%)	2 (0.16)	0	4 (0.79)	6 (0.26)	
Patients with SpA bridging syndesmophytes, N (%)	1 (1.6)	0	2 (9.1)	3 (2.6)	0.167
Quadrants with bridging syndesmophytes per patient	2		2	2	
Patients with SpA changes, N / All patients in the group (%)	14/62 (22.6)	7/32 (21.9)	*11/22 (50.0)* ^*##*^	32/116 (27.6)	0.033
**Spondylophytes**					
Quadrants with spondylophytes, N (%)	308 (25.3)	174 (30.9)	129 (25.5)	611 (26.7)	
Patients with SpA spondylophytes, N (%)	57 (91.9)	28 (87.5)	21 (95.4)	106 (91.4)	0.734
Quadrants with spondylophytes per patient	5.4	6.2	6.1	5.8	
**Bridging spondylophytes**					
Quadrants with bridging spondylophytes, N (%)	46 (3.8)	58 (10.3)	42 (8.3)	146 (6.4)	
Patients with SpA bridging spondylophytes, N (%)	16 (25.8)	17 (53.0)* ^*1*^	11 (50.0)	44 (37.9)	0.036
Quadrants with bridging spondylophytes per patient	2.9	3.4	3.8	3.3	
Patients with degenerative changes N / All patients in the group (%)	58/62 (93.5)	30/32 (93.8)	21/22 (95.5)	109/116 (93.9)	1.000

DISH: Diffuse Idiopathic Skeletal Hyperostosis, axSpA: axial SpondyloArthritis, N/A: Not Applicable

The p-value is related to the simultaneous comparison between the three groups

Italic numbers indicates significant two by two-by-two differences:

^@2^ Significantly higher in Degenerative Disc Disease versus DISH

*^1^ Significantly higher in DISH versus Degenerative Disc Disease

^#1^ Significantly higher in axSpA versus DISH and Degenerative Disc Disease

^##^ Significantly higher in axSpA versus DISH and Degenerative Disc Disease

Percentages are presented in columns

Degenerative changes were also highly prevalent in the three groups. They were identified at the cervical level in 12/20 (60%) patients: 5/9 (55.6%) in DDD, 5/8 (62.5%) in DISH, and 2/3 (66.7%) in axSpA (*p* = 1.000). At the thoracic level, they were identified in 119/128 (93.0%) of patients: 58/66 (87.9%) of DDD, 36/36 (100%) in DISH, and 25/26 (96.1%) of axSpA (*p* = 0.055), with significantly more bridging spondylophytes in DISH (*p* < 0.001). At the lumbar level, they were identified in 109/116 (93.9%) of patients: 58/62 (93.5%) in DDD, 30/32 (93.8%) in DISH, and 21/22 (95.5%) in axSpA, *p* = 1.000, with significantly more bridging spondylophytes in DDD and DISH compared to axSpA (*p* = 0.036).

### Spine MRIs

**Fig. 3:**
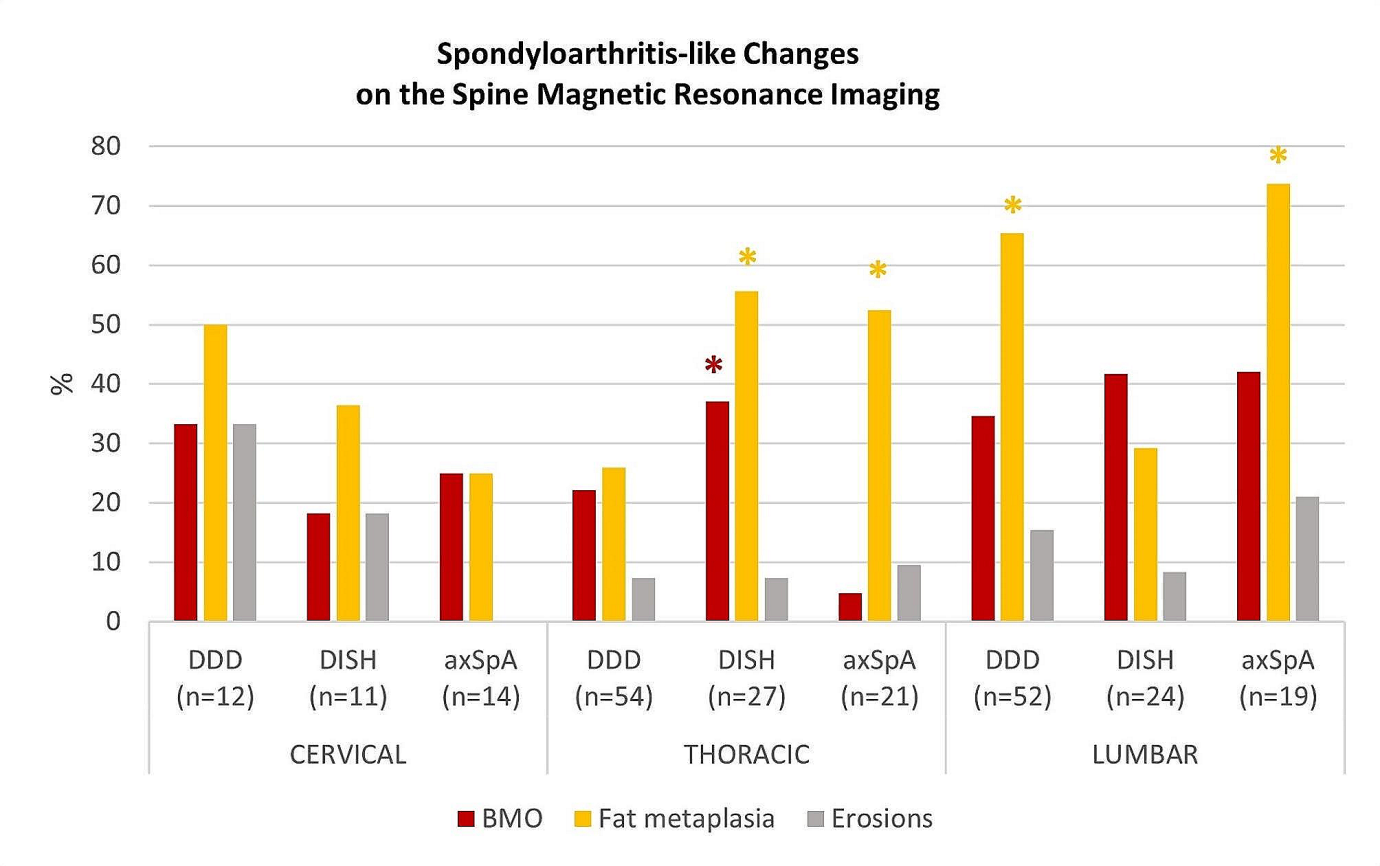
SpA-like changes in the spine MRI in patients with DDD, DISH, and axSpA. axSpA: axial Spondyloarthritis, BMO: Bone Marrow Oedema, DDD: Degenerative Disc Disease, DISH: Diffuse Idiopathic Skeletal Hyperostosis, MRI: magnetic resonance, Imaging, SpA: Spondyloarthritis. *Statistically significant difference: higher prevalence of BMO in DISH vs. axSpA in the thoracic spine; higher prevalence of fat metaplasia in DISH and axSpA vs. DDD in the thoracic spine, in axSpA and DDD vs. DISH in the lumbar spine

Similarly, the three groups had an overlap of inflammatory and degenerative changes in spine MRIs (Table 3; Fig. 3).

**Table 3:** Magnetic resonance imaging lesions (A: Cervical, B: Thoracic, C: Lumbar)

	Degenerative disc disease	DISH	Axial spondyloarthritis	All	*p*-value
**A: Cervical MRI lesions**					
**Number of patients evaluated**	12	11	4	27	
**Number of disc units evaluated**	64	47	15	126	
**Number of quadrants evaluated**	**256**	**188**	**60**	**504**	
**Bone marrow oedema (BMO)**					
Number of quadrants with BMO changes / All quadrants	10 (3.9)	5 (2.7)	3 (5.0)	18 (3.6)	0.940
Number of patients with BMO / All patients (%)	4 (33.3)	2 (18.2)	1 (25.0)	7 (25.9)	0.836
Quadrants with bone marrow oedema per patient	2.5	2.5	3	2.6	
**Erosions**					
Number of quadrants with erosions / All quadrants	4 (1.6)	4 (2.1)	0 (0)	8 (1.6)	0.587
Number of patients with erosions / All patients (%)	4 (33.3)	2 (18.2)	0 (0)	6 (22.2)	0.567
Quadrants with erosions per patient	1	2		1.3	
**Sclerosis**					
Number of quadrants with sclerosis / All quadrants	0 (0)	2 (1.1)	0 (0)	2 (0.4)	0.391
Number of patients with sclerosis / All patients (%)	0	2 (18.2)	0 (0)	2 (7.4)	0.444
Quadrants with sclerosis per patient	0	1		1	
**Fat metaplasia**					
Number of quadrants with fat / All quadrants	14 (5.5)	13 (6.9)	4 (6.7)	31 (6.1)	0.988
Number of patients with fat / All patients (%)	6 (50.0)	4 (36.4)	1 (25.0)	11 (40.71)	0.662
Quadrants with fat metaplasia per patient	2.3	3.2	4	2.8	
**Modic changes**					
Number of discovertebral units with Modic changes / All disc units	4 (6.25)	5 (10.7)	2 (13.3)	11 (8.7)	0.799
Number of patients with Modic changes / All patients (%)	2 (16.7)	4 (33.3)	2 (50)	8 (29.6)	0.232
Discovertebral units with Modic changes per patient	2	1.2	1	1.4	
**Pfirrmann changes**					
Number of discovertebral units with Pfirrmann changes / All disc units	64 (100)	46 (97.9)	15 (100)	125 (99.2)	0.107
Number of patients with Pfirrmann changes / All patients (%)	12 (100)	11 (100)	4 (100)	27 (100)	-
Discovertebral units with Pfirrmann changes per patient	5.3	4.2	3.7	4.6	
**Disc protrusion**					
Number of discovertebral units with disc protrusion / All disc units	19 (29.7)	21 (44.7)	6 (40.0)	46 (36.5)	0.923
Number of patients with disc protrusion / All patients (%)	8 (66.7)	8 (72.7)	2 (50)	18 (66.7)	1.000
Discovertebral units with disc protrusion per patient	2.4	2.6	3	2.5	
**B: Thoracic MRI lesions**					
**Number of patients evaluated**	**54**	**27**	**21**	**102**	
**Number of disc units evaluated**	**241**	**177**	**89**	**507**	
**Number of quadrants evaluated**	**966**	**678**	**356**	**2000**	
**Bone marrow oedema (BMO)**					
Number of quadrants with BMO changes / All quadrants	22 (2.3)	29 (4.3)	4 (1.1)	55 (2.7)	0.077
Number of patients with BMO / All patients (%)	12 (22.2)	10 (37.0)* ^*3*^	1 (4.8)	23 (22.5)	0.024
Quadrants with bone marrow oedema per patient	1.8	2.9	4	2.4	
**Erosions**					
Number of quadrants with erosions / All quadrants	7 (0.7)	2 (0.3)	2 (0.6)	11 (0.5)	0.599
Number of patients with erosions / All patients (%)	4 (7.4)	2 (7.4)	2 (9.5)	8 (7.8)	0.502
Quadrants with erosions per patient	1.7	1	1	1.4	
**Sclerosis**					
Number of quadrants with sclerosis / All quadrants	0 (0)	0 (0)	0 (0)	0 (0)	-
Number of patients with sclerosis / All patients (%)	0 (0)	0 (0)	0 (0)	0 (0)	-
Quadrants with sclerosis per patient					
**Fat metaplasia**					
Number of quadrants with fat / All quadrants	25 (2.6)	56 (8.3)	21 (5.9)	102 (5.1)	< 0.001
Number of patients with fat / All patients (%)	14 (25.9)	*15 (55.6)* ^*1^	*11 (52.4)* ^*#1*^	40 (39.2)	0.014
Quadrants with fat metaplasia per patient	1.7	3.7	1.9	2.5	
**Modic changes**					
Number of discovertebral units with Modic changes / All disc units	5 (2.1)	8 (4.5)	1 (1.1)	14 (2.8)	0.134
Number of patients with Modic changes / All patients (%)	3 (5.6)	5 (18.5)	1 (4.8)	9 (8.8)	0.161
Discovertebral units with Modic changes per patient	1.7	1.6	1	1.6	
**Pfirrmann changes**					
Number of discovertebral units with Pfirrmann changes / All disc units	235 (97.5)	160 (90.4)	83 (93.2)	478 (94.3)	0.081
Number of patients with Pfirrmann changes / All patients (%)	54 (100)	27 (100)	21 (100)	102 (100)	-
Discovertebral units with Pfirrmann changes per patient	4.3	5.9	3.9	4.7	
**Disc Protrusion**					
Number of discovertebral units with disc protrusion / All disc units	31 (12.9)	19 (10.7)	5 (5.6)	55 (11.0)	0.333
Number of patients with disc protrusion / All patients (%)	21 (38.9)	7 (25.9)	4 (19.0)	32 (31.4)	0.221
Discovertebral units with disc protrusion per patient	1.5	2.7	1.2	1.7	
**C: Lumbar MRI lesions**					
**Number of patients evaluated**	**52**	**24**	**19**	**95**	
**Number of disc units evaluated**	**250**	**117**	**95**	**462**	
**Number of quadrants evaluated**	**966**	**678**	**356**	**2000**	
**Bone marrow oedema (BMO)**					
Number of quadrants with BMO changes / All quadrants	34 (3.5)	16 (2.4)	13 (3.6)	63 (3.1)	0.877
Number of patients with BMO / All patients (%)	18 (34.6)	10 (41.7)	8 (42.1)	36 (37.9)	0.769
Quadrants with bone marrow oedema per patient	1.9	1.6	1.6	1.7	
**Erosions**					
Number of quadrants with erosions / All quadrants	9 (0.9)	5 (0.7)	4 (1.1)	18 (0.9)	0.944
Number of patients with erosions / All patients (%)	8 (15.4)	2 (8.3)	4 (21.1)	14 (14.7)	0.502
Quadrants with erosions per patient	1.1	2.5	1	1.3	
**Sclerosis**					
Number of quadrants with sclerosis / All quadrants	1 (0.1)	1 (0.1)	3 (0.8)	5 (0.2)	0.574
Number of patients with sclerosis / All patients (%)	1 (1.9)	1 (4.2)	1 (5.3)	3 (3.2)	1.000
Quadrants with sclerosis per patient	1	1	3	1.7	
**Fat metaplasia**					
Number of quadrants with fat / All quadrants	93 (9.6)	27 (3.9)	43 (12,1)	163 (8.2)	0.192
Number of patients with fat / All patients (%)	*34 (65.4)* ^*@2*^	7 (29.2)	*14 (73.7)* ^*#2*^	55 (57.9)	0.004
Quadrants with fat metaplasia per patient	2.7	3.8	3.1	3.0	
**Modic changes**					
Number of discovertebral units with Modic changes / All disc units	42 (16.8)	9 (7.7)	5 (5.3)	56 (12.1)	0.069
Number of patients with Modic changes / All patients (%)	22 (42.3)	7 (29.2)	3 (15.8)	32 (33.7)	0.097
Discovertebral units with Modic changes per patient	1.9	1.3	1.7	1.7	
**Pfirrmann changes**					
Number of discovertebral units with Pfirrmann changes / All disc units	250 (100)	102 (87.2)	93 (97.9)	445 (96.3)	0.021
Number of patients with Pfirrmann changes / All patients (%)	*52 (100)* ^*@2*^	21 (87.5)	19 (100)	92 (96.8)	0.022
Discovertebral units with Pfirrmann changes per patient	4.8	4.8	4.9	4.8	
**Disc protrusion**					
Number of discovertebral units with disc protrusion / All disc units	193 (77.2)	76 (64.9)	52 (54.7)	321 (69.5)	0.031
Number of patients with disc protrusion / All patients (%)	50 (96.2)	20 (83.3)	17 (89.5)	87 (91.6)	0.102
Discovertebral units with disc protrusion per patient	3.8	3.8	3.1	3.7	

DISH: Diffuse Idiopathic Skeletal Hyperostosis

Modic changes were defined as type I, II, or III

Pfirrmann changes were defined as grade II, III, IV, and V

Disc protrusion was defined as grade I or II

The p-value is related to the simultaneous comparison between the three groups

Italic number indicates significant two by two-by-two differences:

^#1^ Significantly higher in axial Spondyloarthritis versus Degenerative Disc Disease

*^1^ Significantly higher in DISH versus Degenerative Disc Disease

*^3^ Significantly higher in DISH versus axial Spondyloarthritis

^@2^ Significantly higher in Degenerative Disc Disease versus DISH

^#2^ Significantly higher in axial Spondyloarthritis versus DISH

Percentages are presented in columns

In the cervical spine, BMO was present in 4/12 (33.3%) in DDD, 2/11 (18.2%) in DISH, and 1/4 (25.0%) in axSpA patients (*p* = 0.836). At least one degenerative change was identified in 100% of the patients in the three groups, and these were mostly Pfirrmann changes.

In the thoracic spine, BMO was present in 12/54 (22.2%) in DDD, 10/27 (37%) in DISH, and 1/21 (4.8%) in axSpA patients (*p* = 0.024). Fat metaplasia was observed significantly more frequently in patients with DISH (15/27 (55.6%)) and axSpA (11/21 (52.4%)), compared to DDD (14/54 (25.9%)), *p* = 0.014.

In the lumbar spine, BMO was observed in 18/52 (34.6%) in DDD, 10/24 (41.7%) in DISH, and 8/19 (42.1%) in axSpA patients (*p* = 0.769). Fat metaplasia was significantly higher in patients with axSpA (14/19 (73.7%)) and DDD (34/52 (65.4%)), compared to patients with DISH (7/24 (29.2%)), *p* = 0.004. Degenerative changes were present among the three groups, with 87.5–100% of patients presenting at least one degenerative change.

### Sacroiliac joints X-rays

SIJ X-rays were available for 117 patients in total (61/71 DDD, 31/38 DISH, and 25/27 axSpA patients). As per the New York criteria, they were positive for sacroiliitis in 48.7% (76.0% in axSpA, 47.5% in DDD, and 29.0% in DISH, *p* = 0.002) (Supplementary Table 1).

### MRIs of the SIJ

SIJ MRIs were available for 30 patients (11/71 DDD, 10/38 DISH, and 9/27 axSpA). BMO on any SIJ quadrant was present in 12 patients (2/11 DDD, 7/10 DISH patients, and 3/9 axSpA *p* = 0.052). Fat deposition on any SIJ quadrant was present in 7 patients (3/11 DDD, 0/10 DISH patients, and 4/9 axSpA, *p* = 0.076). Erosions were present on any SIJ quadrant in 3 patients (2/11 DDD, 0/10 DISH patients, and 1/9 axSpA, *p* = 0.621) (Supplementary Table 2).

## Discussion

This cross-sectional study with real-life data describes a significant overlap of inflammatory and degenerative features on spinal imaging among patients with chronic LBP and diagnosed with DDD, DISH, and axSpA.

On CR, SpA-like changes were identified in patients with DISH in the thoracic spine in 28% and the lumbar spine in 22% in our study. Notably, in the thoracic spine, 3/36 patients with DISH (8.3%) had bridging syndesmophytes. In a recent study using whole spine computed tomography on 111 DISH and axSpA [27], 11% of patients with DISH had smooth-type anterior bony bridging.

On MRI, we identified SpA-like lesions in patients with DISH in all three spinal segments: BMO in 18% in the cervical, 37% in the thoracic, and 42% in the lumbar spine. Also, FL were identified in more than half of the thoracic spines. These findings align with those of Latourte et al., who reported that 58% of 53 patients with DISH met the ASAS definition of a spine MRI suggestive of axSpA, and 67% had at least one FL [16].

Conversely, in the present analysis, patients with axSpA had degenerative spine changes in 67%, 96%, and 96% on CR of the cervical, thoracic, and lumbar spine, respectively. In a study from the DESIR cohort [14], in 648 patients, degenerative lesions were found in about 70% of patients with axSpA.

Furthermore, we identified significant overlaps in the SIJ as well. On CR, SIJ were positive for SpA-like sacroiliitis according to the NY criteria in 48% of patients with DDD and 29% of those with DISH. Similarly, in a recent study on 111 DISH and axSpA [27], 63% of patients with DISH had a partial or complete SIJ fusion. In another recent study on 90 axSpA patients aged 65 years and 90 age- and sex-matched controls [28], joint space narrowing, erosion, and sclerosis were present in controls, albeit with lower rates than patients with axSpA. In that study by Fakih et al., 58% of axSpA patients had complete bilateral ankylosis, while one case (1.1%) of bilateral ankylosis was found in the control group in the context of severe DISH.

Despite the overlap, in our study, the total score average for both SIJs was significantly higher in patients with axSpA (4.50 [2.13]) compared to DDD (3.51 [1.51]) and DISH (2.53 (1.83)), *p* < 0.001.

On SIJ MRI, the current study identified SpA-like BMO in 70% of patients with DISH on any quadrant, bilaterally in 20%. However, FL and erosions were not identified in patients with DISH. Thus, the simultaneous presence of both inflammatory and structural changes, particularly FL and erosions, was more in favor of the axSpA diagnosis vs. DISH. In a study on 309 consecutive patients with chronic back pain diagnosed with axSpA (175) or non-SpA (134), SIJ quadrants with BMO and erosions were significantly more frequent in axSpA vs. non-SpA patients independent of age, while this difference was seen for FL only in patients ≥ 50 years [15]. Also, in a retrospective cross-sectional study of 485 non-axSpA patients, FL were identified in 50% of subjects < 45 years old and 94% of patients > 75 years old [29]. Nevertheless, SIJ erosions were rarely identified in non-axSpA individuals, as 0.6% of patients < 45 years old and 2.6% of the entire study population exhibited this feature, with no detectable age-dependent increase. Sclerosis and spondylophytes were detected in 13.7% and 37.0% of patients, respectively. Similarly, only 3.3% of DISH patients had > 3 erosions on the SIJ in the study by Latourte et al. [16], where 6/53 (15.8%) patients with an available SIJ MRI had sacroiliitis according to ASAS criteria.

The current study’s limitations are mostly related to its real-life setting: patients were diagnosed for disease categories according to the judgment of the treating rheumatologist, and SIJ imaging and HLA-B27 were available only for a proportion of patients. In fact, the relatively small number of patients, especially in some study categories, reflects the real-life setting and the prescription of imaging and laboratory tests for some difficult-to-diagnose patients. In addition, circular reasoning might have affected the study results. Finally, classification bias might occur, as some patients might be misclassified into one of the three groups, particularly in patients with PsA who might have axial involvement. However, in patients with DISH, the association with PsA is limited to 5%.

Despite these limitations, the current study provides valuable insights into the diagnostic challenges that rheumatologists face when consulting older patients with chronic LBP and supports the minimization of overdiagnosis of axSpA. Unlike most studies focused on SIJ, this study is among the few that provided detailed data about spine imaging, particularly an analysis of the three spine levels: cervical, thoracic, and lumbar. In addition, previous studies have demonstrated the reliability and reproducibility of the scoring method used for the assessment of spinal imaging [30–32].

Future imaging techniques and image analysis may help differentiate between axSpA and mimics. For instance, a study by Terrier et al. [33] reported that the subchondral bone attenuation coefficient of the sacroiliac margins on CT scans may help differentiate axSpA from osteocondensans ilii. Also, predictive diagnostic models, including the patient’s age, body mass index and whole-body MRI, were studied in 48 patients and showed promising diagnostic properties. However, its generalizability might be challenging due to the lack of universal availability of whole-body MRI [34].

## Conclusions

In a real-life cohort of older patients referred to a tertiary center for low back pain, significant overlap occurred between inflammatory and degenerative features on spine imaging among DISH, DDD, and axSpA. Particularly, axSpA-related spine X-ray features were found in one-fourth of patients with DISH, and MRI BMO was found in one-third. Therefore, careful interpretation of imaging data should be conducted in practice, as it always should be integrated into a holistic diagnostic approach.

### Electronic supplementary material

Below is the link to the electronic supplementary material.

Supplementary Material 1

## Data Availability

No datasets were generated or analysed during the current study.

## Abbreviations

axSpA: Axial spondyloarthritis
BASDAI: Bath Ankylosing Spondylitis Disease Activity Index
BASFI: Bath Ankylosing Spondylitis Functional Index
bDMARDs: Biological disease-modifying anti-rheumatic drugs
BMI: Body Mass Index
BMO: Bone marrow oedema
CR: Conventional radiographs
CRP: C reactive protein
csDMARDs: Conventional synthetic disease-modifying anti-rheumatic drugs
DC: Degenerative changes
DDD: Degenerative disc disease
DISH: Diffuse idiopathic skeletal hyperostosis
ESR: Erythrocyte Sedimentation rate
FL: Fat lesions
HLAB27: Human Leucocyte Antigen B27
IQR: Interquartile range
LBP: Low back pain
MRI: Magnetic resonance imaging
NRS: Numerical rating scale
NSAIDs: Non-steroidal anti-inflammatory drugs
RA: Rheumatoid arthritis
PsA: Psoriatic arthritis
SIJ: Sacroiliac joints
SpA: Spondyloarthritis
