# Supplementary material for: Significant overlap of inflammatory and degenerative features on imaging among patients with degenerative disc disease, diffuse idiopathic skeletal hyperostosis and axial spondyloarthritis: a real-life cohort study
Source: Arthritis Res Ther. 2024 Aug 3;26:147. doi: 10.1186/s13075-024-03359-w (PMC11297750; doi:10.1186/s13075-024-03359-w)
Supplement: Supplementary file 1 — Supplementary Material 1 [file 13075_2024_3359_MOESM1_ESM.docx]

**Supplementary Table 1. X-rays of the sacroiliac joints in the three groups**

|  | **Degenerative**  **Spine Disease** | **DISH** | **Axial Spondyloarthritis** | **All** | **p-value** |
| --- | --- | --- | --- | --- | --- |
| Number of X-rays available/ All patients | 61/71 | 31/38 | 25/27 | 117/136 |  |
| Total Score both SIJ*, average [SD] | 3.51 [1.51]^@2^ | 2.53 (1.83) | 4.50 [2.13]^##^ | 3.43 [1.86] | <0.001 |
| X-ray positive for sacroiliitis**, N (%) | 29 (47.5) | 9 (29.0) | 19 (76.0)^##^ | 57 (48.7) | 0.002 |

*Sum of the scores of the two sacroiliac joints, each score ranging from 0 (normal) to 4 (ankylosis), total score ranging from 0 to 8

**Sacroiliitis is defined by having at least a grade 2 bilaterally or grade 3 unilaterally as per the New York criteria

The p-value is related to the simultaneous comparison between the three groups.

^#1^ Significantly higher in axSpA versus DDD

^##^ Significantly higher in axSpA versus DISH and Degenerative Disc Disease

^@2^ Significantly higher in Degenerative Disc Disease versus DISH

DISH: Diffuse Idiopathic Skeletal Hyperostosis, SIJ: Sacroiliac Joints
